# Supplementary material for: The Effect of Environmental Factors on the Nutrition of European Beech (Fagus sylvatica L.) Varies with Defoliation
Source: Plants (Basel). 2022 Dec 30;12(1):168. doi: 10.3390/plants12010168 (PMC9823833; doi:10.3390/plants12010168)
Supplement: Supplementary file 1 [file plants-12-00168-s001.zip › plants-2036165-supplementary.pdf]

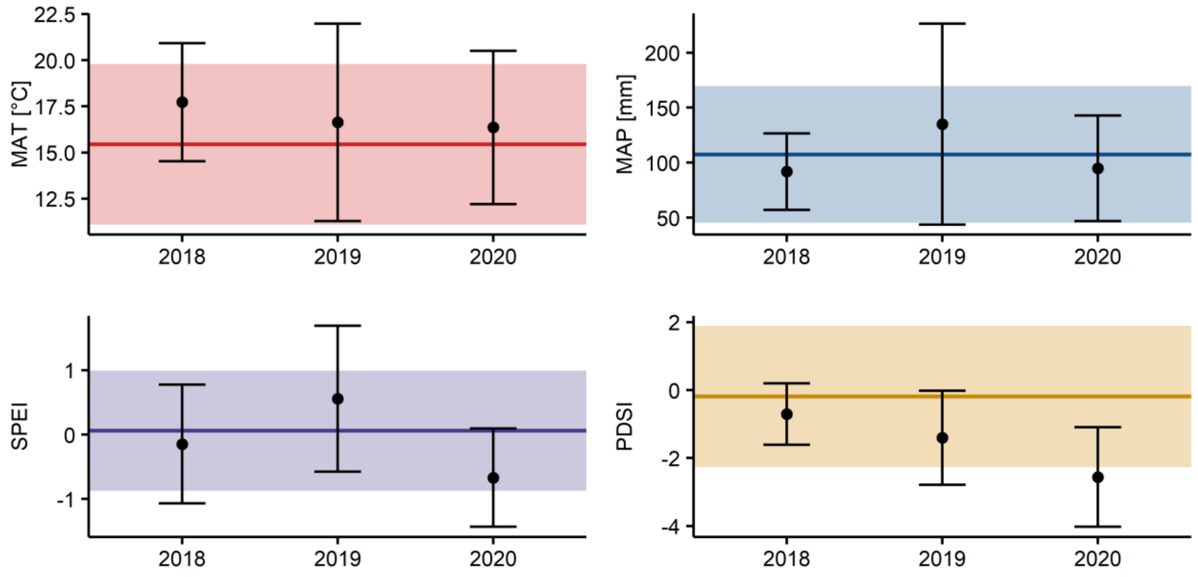

Figure S1. Comparison of study area climate properties during the vegetation period with the climate normal (1980 – 2011) of the vegetation period (March -September). Mean value (dot) and standard deviation (vertical bars) of Mean Annual Temperature (MAT), Mean Annual Precipitation (MAP), Standardized Precipitation Evapotranspiration Index (SPEI) and Palmer Drought Severity Index (PDSI) are shown for each sampling year (2018 – 2020). Climate normal mean values and standard deviations for each parameter are represented by a horizontal line and shaded area, respectively.

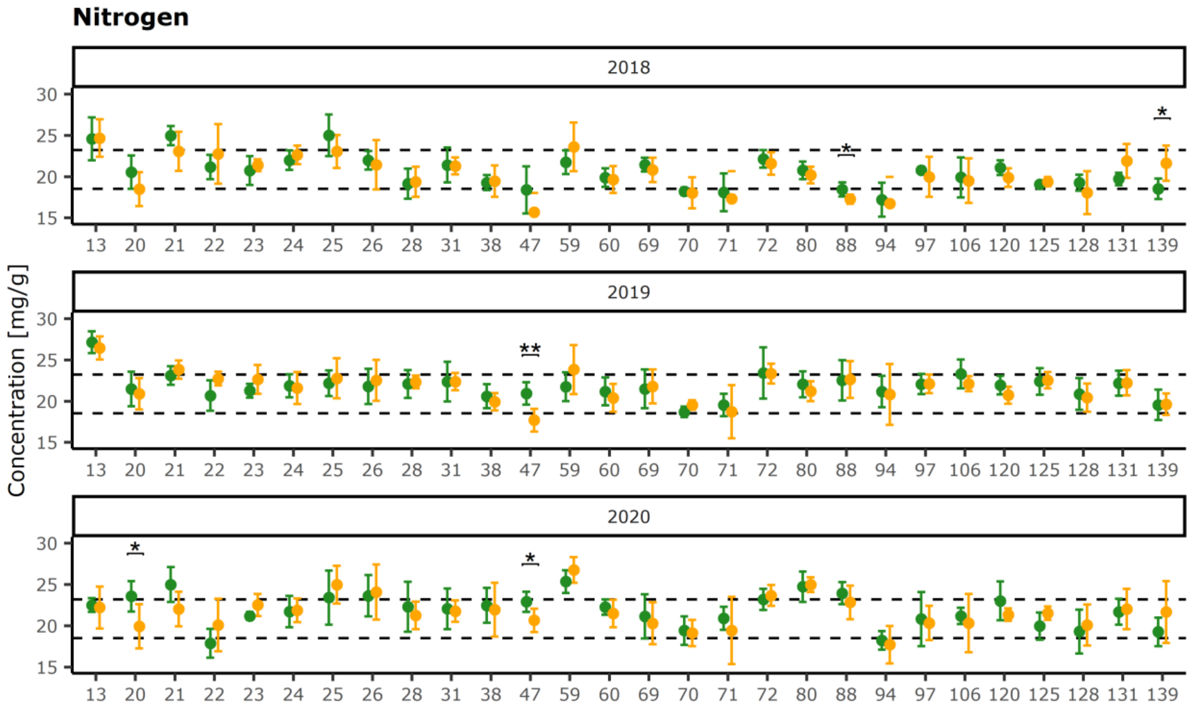

Figure S2. Foliar nitrogen concentrations on research plots (plot number on x-axis), mean values (dot) and confidence intervals (vertical bars) for LD (green colour) and HD trees (orange colour). Dotted lines represent lower and upper critical value for normal range of foliar N concentrations for common beech according to Mellert and Goettlein (2012). Only significant differences in foliar concentrations between LD and HD categories determined by the independent samples *t*-test are indicated ($^{*}p < 0.05$, $^{**}p < 0.01$, $^{***}p < 0.001$).

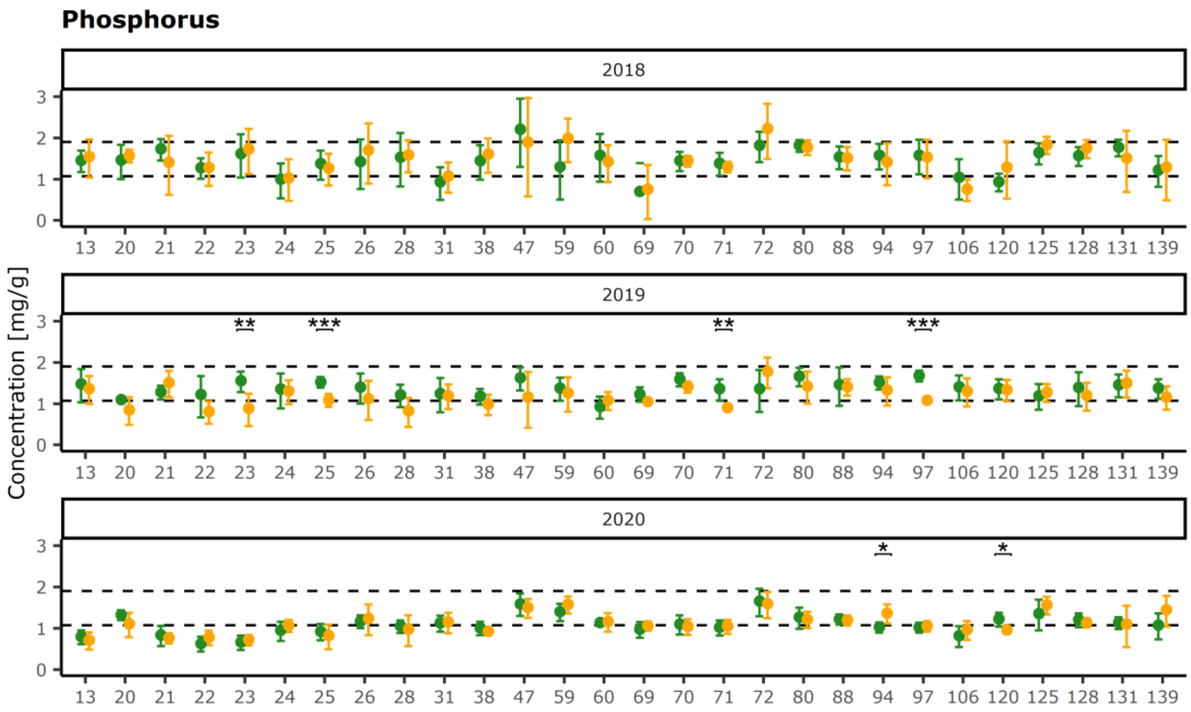

Figure S3. Foliar phosphorus concentrations on research plots (plot number on x-axis), mean values (dot) and confidence intervals (vertical bars) for LD (green colour) and HD trees (orange colour). Dotted lines represent lower and upper critical value for normal range of foliar P concentrations for common beech according to Mellert and Goettlein (2012). Only significant differences in foliar concentrations between LD and HD categories determined by the independent samples *t*-test are indicated ($^{*}p < 0.05$, $^{**}p < 0.01$, $^{***}p < 0.001$).

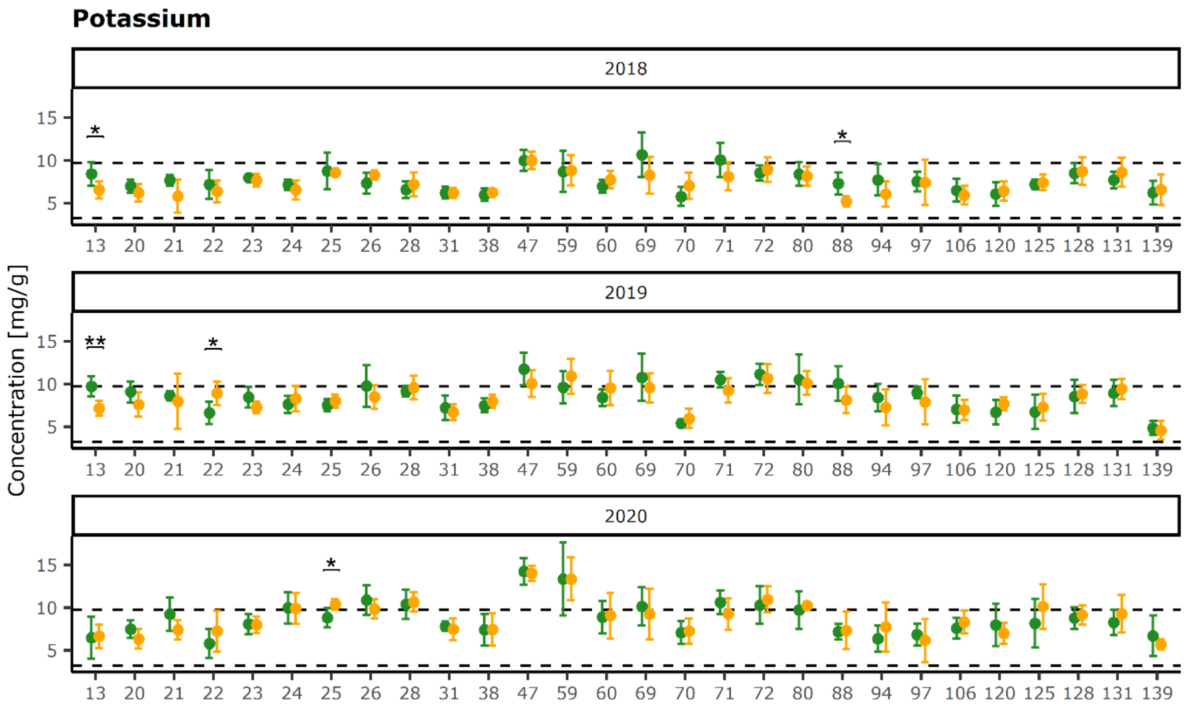

Figure S4. Foliar potassium concentrations on research plots (plot number on x-axis), mean values (dot) and confidence intervals (vertical bars) for LD (green colour) and HD trees (orange colour). Dotted lines represent lower and upper critical value for normal range of foliar K concentrations for common beech according to Mellert and Goettlein (2012). Only significant differences in foliar concentrations between LD and HD categories determined by the independent samples *t*-test are indicated ($^{*}p < 0.05$, $^{**}p < 0.01$, $^{***}p < 0.001$).

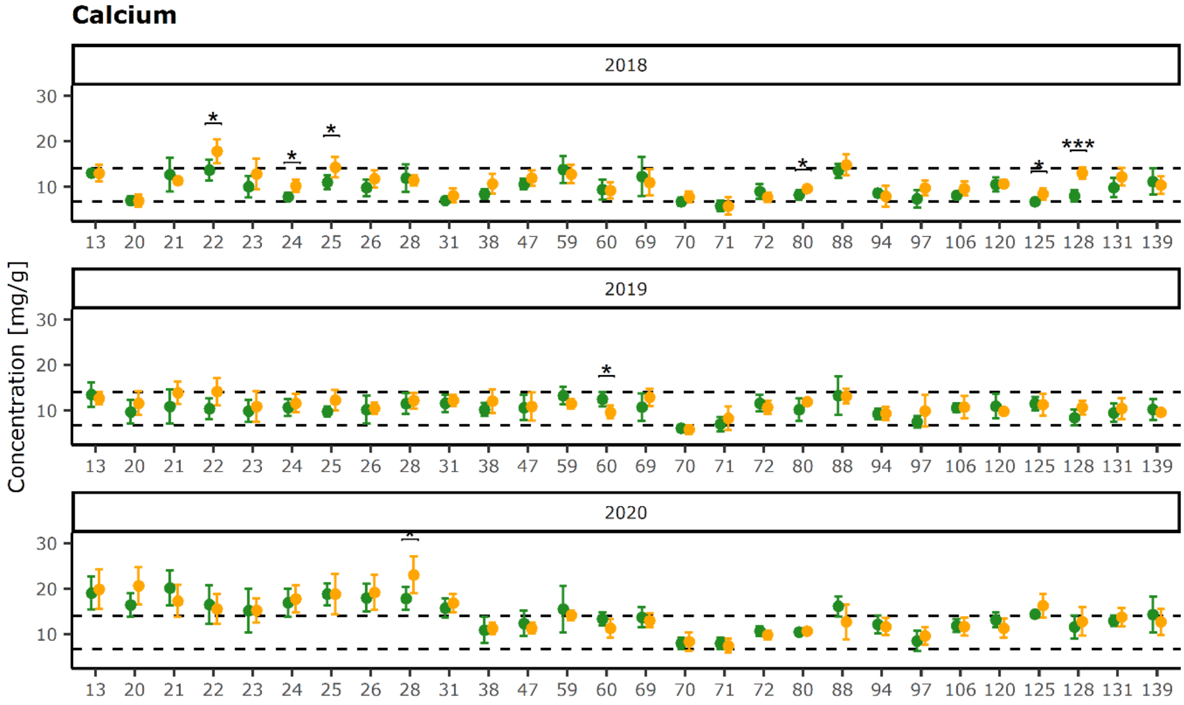

Figure S5. Foliar calcium concentrations on research plots (plot number on x-axis), mean values (dot) and confidence intervals (vertical bars) for LD (green colour) and HD trees (orange colour). Dotted lines represent lower and upper critical value for normal range of foliar Ca concentrations for common beech according to Mellert and Goettlein (2012). Only significant differences in foliar concentrations between LD and HD categories determined by the independent samples *t*-test are indicated (*p < 0.05, **p < 0.01, ***p < 0.001).

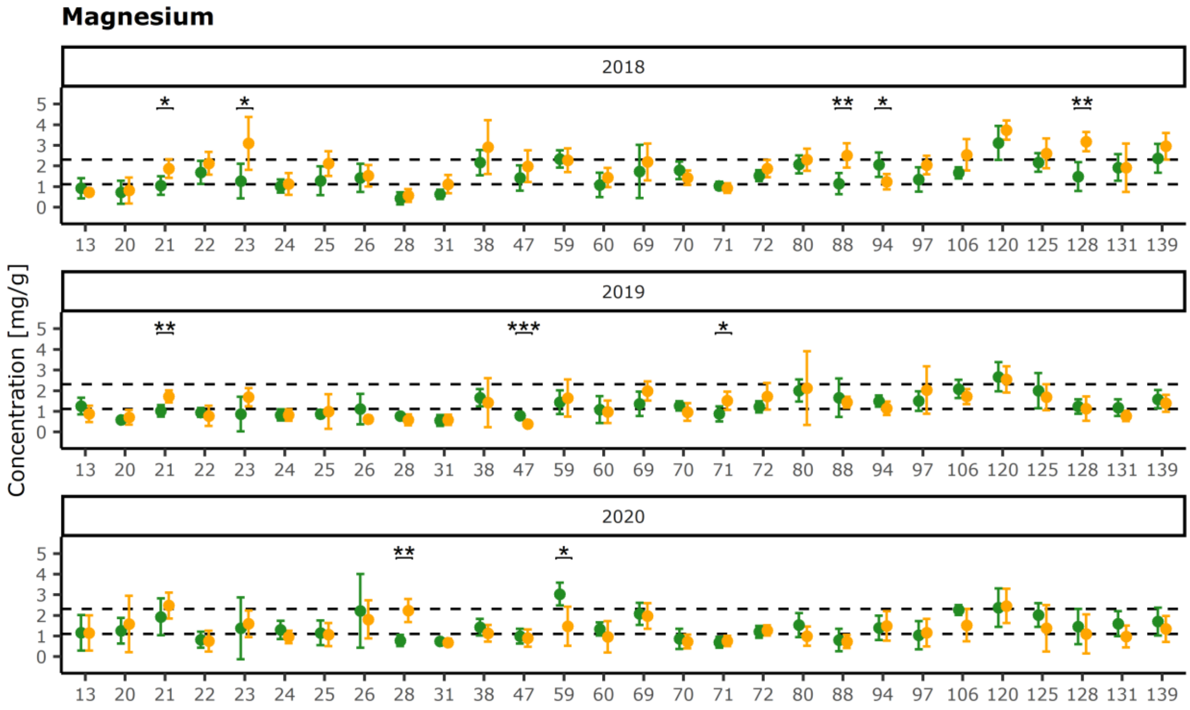

Figure S6. Foliar magnesium concentrations on research plots (plot number on x-axis), mean values (dot) and confidence intervals (vertical bars) for LD (green colour) and HD trees (orange colour). Dotted lines represent lower and upper critical value for normal range of foliar Mg concentrations for common beech according to Mellert and Goettlein (2012). Only significant differences in foliar concentrations between LD and HD categories determined by the independent samples *t*-test are indicated (*p < 0.05, **p < 0.01, ***p < 0.001).

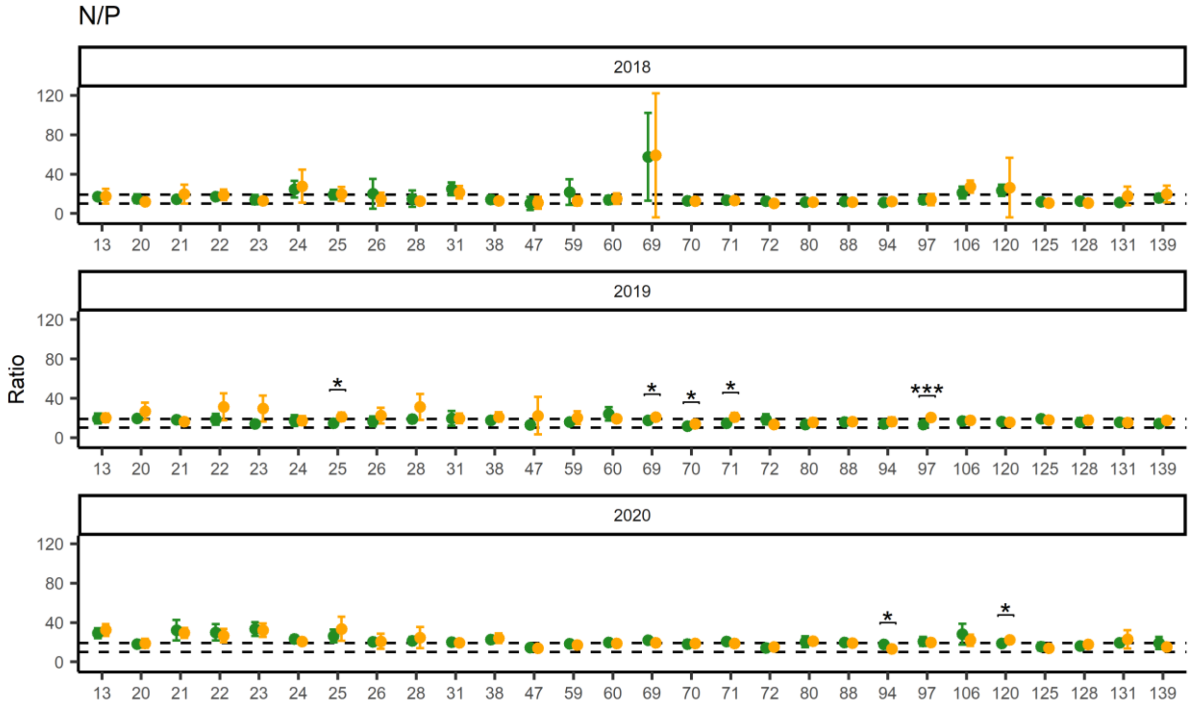

Figure S7. Foliar N/P ratios on research plots (plot number on x-axis), mean values (dot) and confidence intervals (vertical bars) for LD (green colour) and HD trees (orange colour). Dotted lines represent lower and upper critical value for normal range of foliar N/P ratios for common beech according to Mellert and Goettlein (2012). Only significant differences in foliar ratios between LD and HD categories determined by the independent samples *t*-test are indicated ($^{*}p < 0.05$, $^{**}p < 0.01$, $^{***}p < 0.001$).

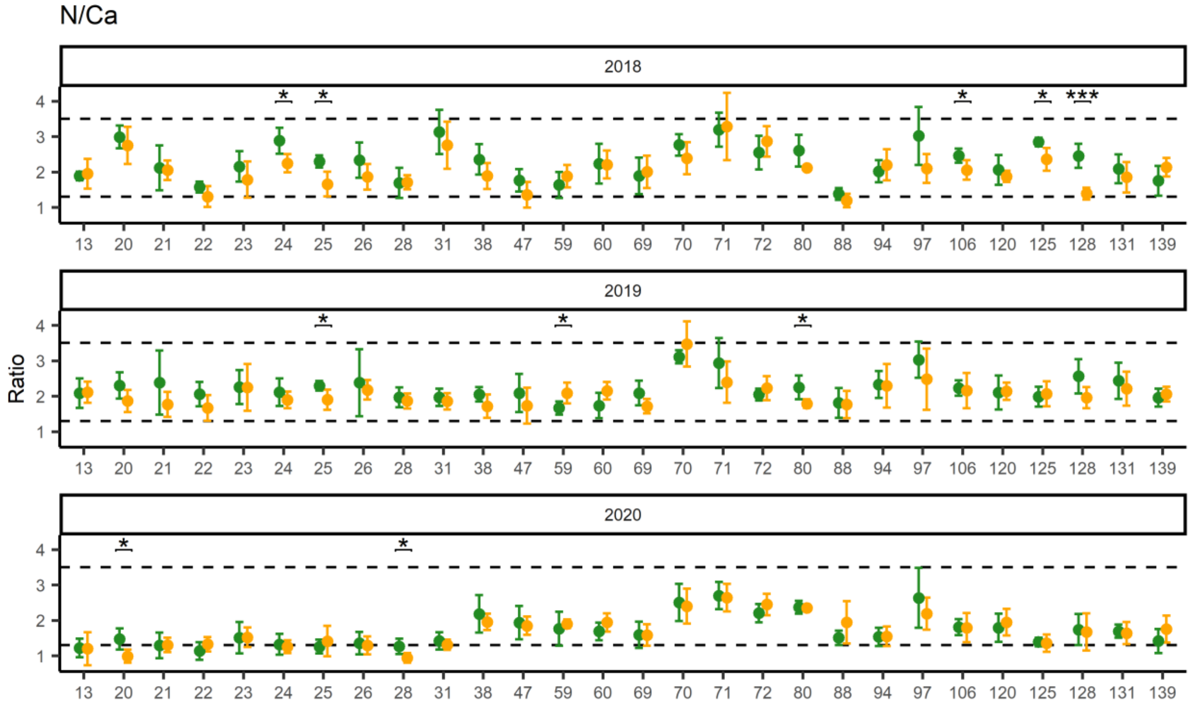

Figure S8. Foliar N/Ca ratios on research plots (plot number on x-axis), mean values (dot) and confidence intervals (vertical bars) for LD (green colour) and HD trees (orange colour). Dotted lines represent lower and upper critical value for normal range of foliar N/Ca ratios for common beech. Only significant differences in foliar ratios between LD and HD categories determined by the independent samples *t*-test are indicated ($^{*}p < 0.05$, $^{**}p < 0.01$, $^{***}p < 0.001$).

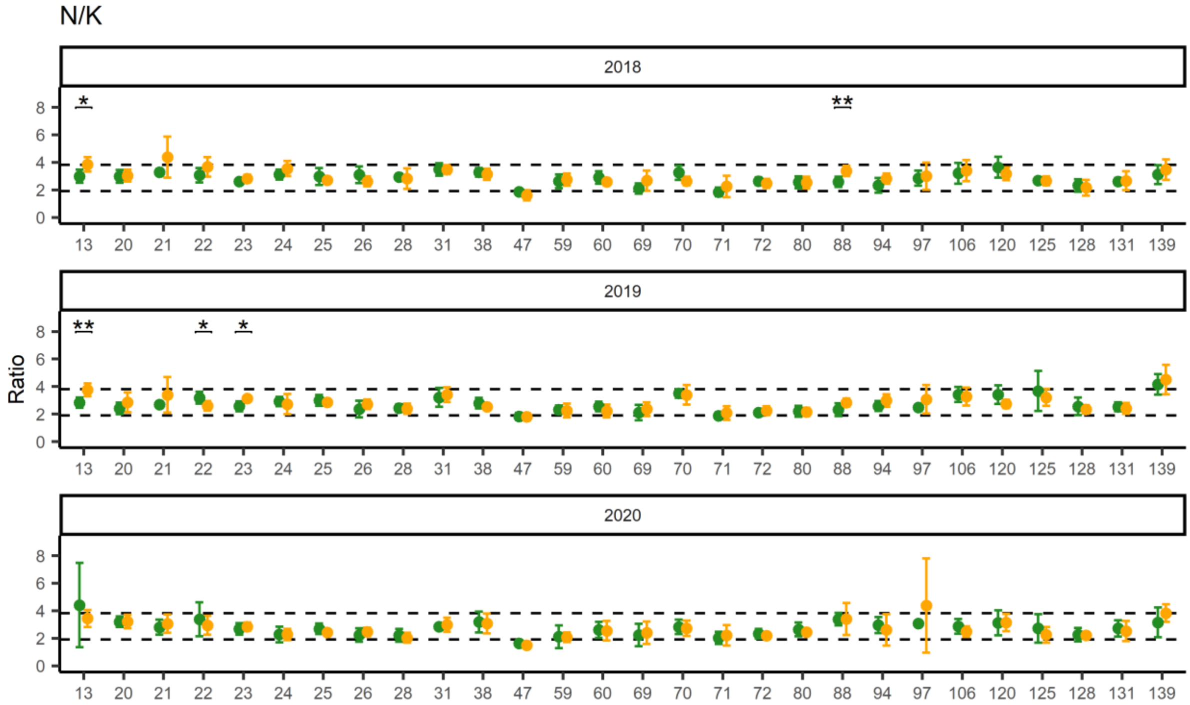

Figure S9. Foliar N/K ratios on research plots (plot number on x-axis), mean values (dot) and confidence intervals (vertical bars) for LD (green colour) and HD trees (orange colour). Dotted lines represent lower and upper critical value for normal range of foliar N/K ratios for common beech. Only significant differences in foliar ratios between LD and HD categories determined by the independent samples *t*-test are indicated ($^{*}p < 0.05$, $^{**}p < 0.01$, $^{***}p < 0.001$).

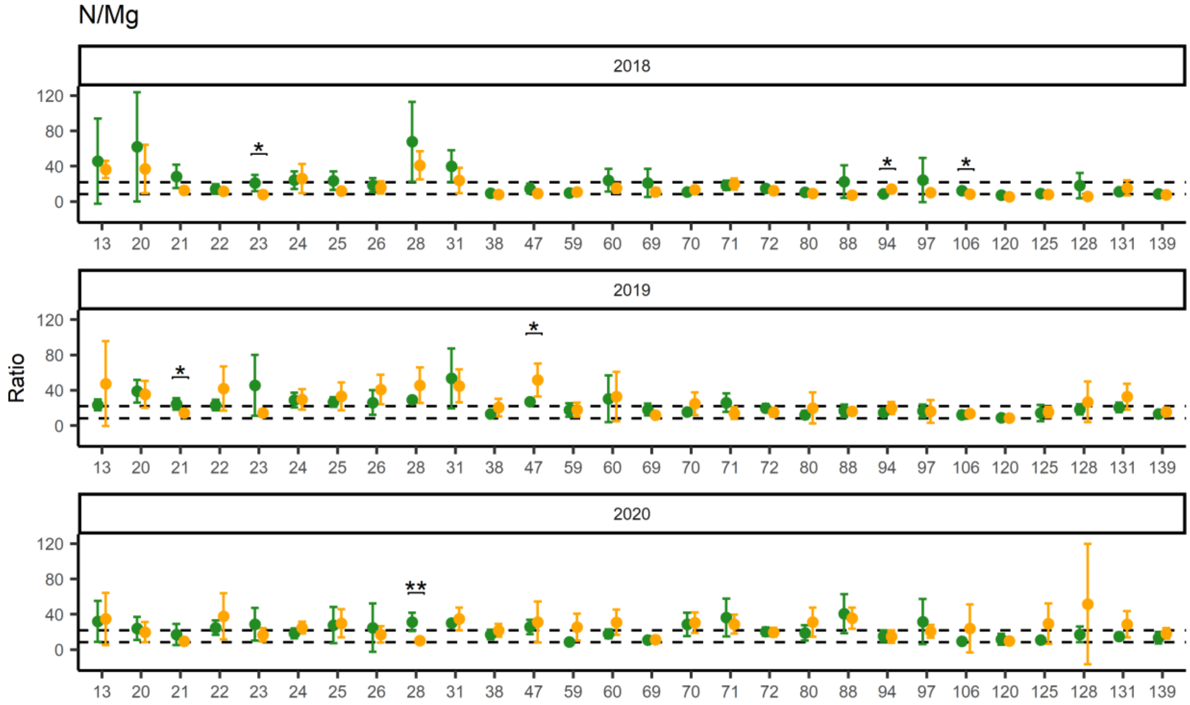

Figure S10. Foliar N/Mg ratios on research plots (plot number on x-axis), mean values (dot) and confidence intervals (vertical bars) for LD (green colour) and HD trees (orange colour). Dotted lines represent lower and upper critical value for normal range of foliar N/Mg ratios for common beech. Only significant differences in foliar ratios between LD and HD categories determined by the independent samples *t*-test are indicated ($^{*}p < 0.05$, $^{**}p < 0.01$, $^{***}p < 0.001$).

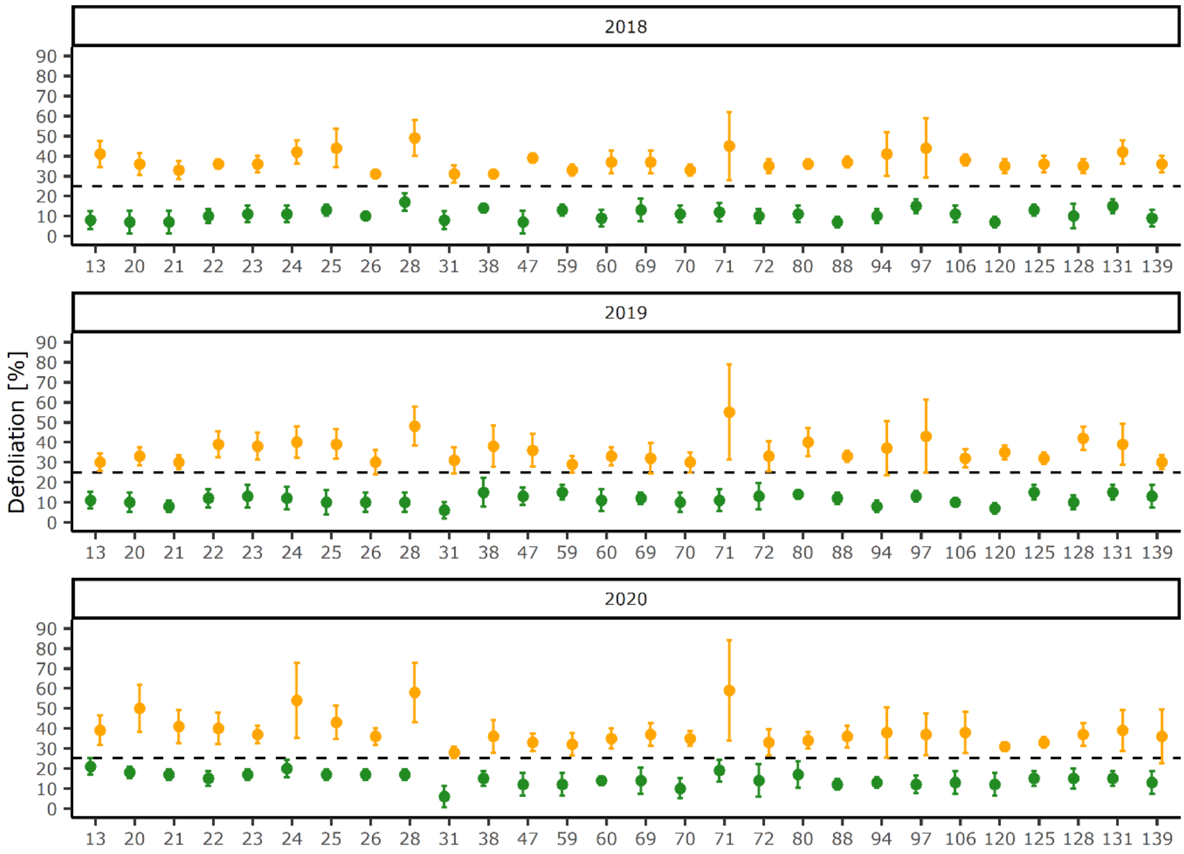

Figure S11. Defoliation of common beech on research plots, mean values (dot) and standard deviation (vertical bars) for LD (green colour) and HD trees (orange colour). Dotted line represents the 25%-defoliation threshold.

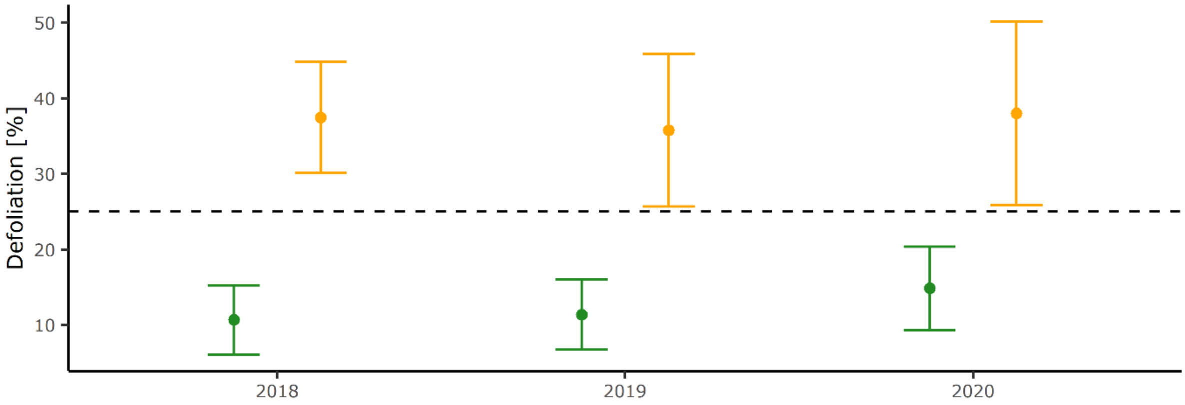

Figure S12. Defoliation of common beech on all plots during the study period, mean values (dot) and standard deviation (vertical bars) for LD (green colour) and HD trees (orange colour). Dotted line represents the 25%-defoliation threshold
